# Ex vivo propagation in a novel 3D high-throughput co-culture system for multiple myeloma

**DOI:** 10.1007/s00432-021-03854-6

**Published:** 2022-01-24

**Authors:** Johannes M. Waldschmidt, Stefan J. Fruttiger, Dagmar Wider, Johannes Jung, Andreas R. Thomsen, Tanja N. Hartmann, Justus Duyster, Martin J. Hug, Kareem A. Azab, Manfred Jung, Ralph Wäsch, Monika Engelhardt

**Affiliations:** 1 Department of Internal Medicine I, Faculty of Medicine and Medical Center, University of Freiburg, Hugstetterstr. 53, 79106 Freiburg, Germany; 2 Comprehensive Cancer Center Freiburg (CCCF), Freiburg University Medical Center, Freiburg, Germany; 3 Pharmacy, Freiburg University Medical Center, Freiburg, Germany; 4 Department of Radiation Oncology, Freiburg University Medical Center, Freiburg, Germany; 5 Institute of Pharmaceutical Sciences, University of Freiburg, Freiburg, Germany; 6 Department of Radiation Oncology, Washington University, St. Louis, MO USA

**Keywords:** Multiple myeloma, Drug discovery, In vitro modeling, Bone marrow microenvironment, Bortezomib, Auranofin

## Abstract

**Purpose:**

Multiple myeloma (MM) remains an incurable hematologic malignancy which ultimately develops drug resistance and evades treatment. Despite substantial therapeutic advances over the past years, the clinical failure rate of preclinically promising anti-MM drugs remains substantial. More realistic in vitro models are thus required to better predict clinical efficacy of a preclinically active compound.

**Methods:**

Here, we report on the establishment of a conical agarose 3D co-culture platform for the preclinical propagation of primary MM cells ex vivo. Cell growth was compared to yet established 2D and liquid overlay systems. MM cell lines (MMCL: RPMI-8226, U266, OPM-2) and primary patient specimens were tested. Drug sensitivity was examined by exploring the cytotoxic effect of bortezomib and the deubiquitinase inhibitor auranofin under various conditions.

**Results:**

In contrast to 2D and liquid overlay, cell proliferation in the 3D array followed a sigmoidal curve characterized by an initial growth delay but more durable proliferation of MMCL over 12 days of culture. Primary MM specimens did not expand in ex vivo monoculture, but required co-culture support by a human stromal cell line (HS-5, MSP-1). HS-5 induced a > fivefold increase in cluster volume and maintained long-term viability of primary MM cells for up to 21 days. Bortezomib and auranofin induced less cytotoxicity under 3D vs. 2D condition and in co- vs. monoculture, respectively.

**Conclusions:**

This study introduces a novel model that is capable of long-term propagation and drug testing of primary MM specimens ex vivo overcoming some of the pitfalls of currently available in vitro models.

**Supplementary Information:**

The online version contains supplementary material available at 10.1007/s00432-021-03854-6.

## Introduction

Multiple myeloma (MM) is a plasma cell disorder with an expected number of 34,920 new cases in the United States in 2021 (American Cancer Society 2021). It is characterized by end-organ damage, such as anemia, hypocalcemia, renal insufficiency, and bone lesions (Kyle and Rajkumar 2009). Overall response rates to induction therapy have substantially improved over the past decades and are nowadays consistently > 90%, with a pooled 5 year overall survival (OS) of 54% across all age and risk groups (American Cancer Society 2019). Despite such substantial progress, MM remains an incurable disease that ultimately develops resistance and evades treatment. Novel therapeutic strategies are thus needed, especially for patients with triple-refractory MM, i.e., a disease state that is refractory to bortezomib, lenalidomide, and anti-CD38 antibodies (Varga et al. 2020). The failure rate of clinical trials, however, remains sizeable as exemplified by the limited clinical activity of the anti-IL-6 antibody siltuximab (Voorhees et al. 2013), the anti-BAFF antibody tabalumab (Raje et al. 2016, 2017), and the anti-CXCR4 antibody ulocuplumab (Ghobrial et al. 2020) in patients with relapsed/ refractory MM (RRMM). More realistic preclinical models are thus urgently required to better predict later clinical success in patients.

In this study, we report on the establishment of a three-dimensional (3D) co-culture platform for the preclinical propagation of primary MM cells ex vivo. As a basis for our model, we used a conical agarose 3D microwell array (3D CoSeedis^™^) which so far has been developed for solid tumors (Thomsen et al. 2017). MM cells are cultured in conical microwells formed of a non-adherent agarose matrix with bone marrow (BM)-derived stromal cells plated on the base of each plate. This distant co-culture model allows for the diffusion of soluble cytokines between BM stromal and MM cells. It stimulates aggregate formation of primary patient cells and allows for co-culture with compounds of the BM stroma for up to 21 days ex vivo*.* To examine the applicability of our model, we exemplified drug testing in comparison to yet established in vitro models by assessing the cytotoxic efficacy of the proteasome inhibitor bortezomib under 3D vs. 2D conditions and in co- vs. monoculture. We recapitulated our findings by testing auranofin, an inhibitor of proteasome-associated deubiquitinases (DUB) with reported preclinical activity in MM (Nakaya et al. 2011).

In addition to reducing cost and time for more realistic preclinical drug screening, the here established 3D model holds the potential to perform drug testing in primary patient samples and to predict individual treatment responses ex vivo in *real-time.*

## Methods

### Tumor cell lines

The MM cell lines (MMCL) RPMI-8226, U266, OPM-2, and the human stromal cell line HS-5 were obtained from commercial sources (DSMZ, Braunschweig, Germany). MSP-1 was provided by Dr. K. Azab (Washington University, St. Louis, MO) (de la Puente et al. 2016). All cell lines were cultured as previously reported (Udi et al. 2013; Waldschmidt et al. 2017). Authentication of cell lines was performed using DNA fingerprinting with small tandem repeat (STR) profiling. All cell lines were tested and found to be negative for mycoplasma contamination.

### Primary samples

BM samples from six patients with untreated or relapsed/refractory MM were included in this study. The mean BM infiltration rate at the time of sample acquisition was 83% (range 80–90%). All patients provided written informed consent in accordance with local ethical standards (UKF protocol 212-16) and the Declaration of Helsinki. Mononuclear cells were isolated by density gradient centrifugation (Ficoll), frozen, and stored in liquid nitrogen until further use. Given the high percentage of histologically determined BM infiltration rates at the time of sample acquisition, thawed MM cells were further enriched using MACS technology-based bead selection for CD117 (Miltenyi Biotec). This approach helped to avoid interference with therapeutically relevant antigens (CD38, SLAMF7, and BCMA) (Schmidt-Hieber et al. 2011) and was independent of the well-described phenomenon of CD138 downregulation after storage at − 80 °C (Frigyesi et al. 2014). To allow for comparability, a density of 5 × 10^5^ cells per mL was used in all experiments for primary MM cells.

### 3D CoSeedis^™^ conical agarose microwell array

**Fig. 1:**
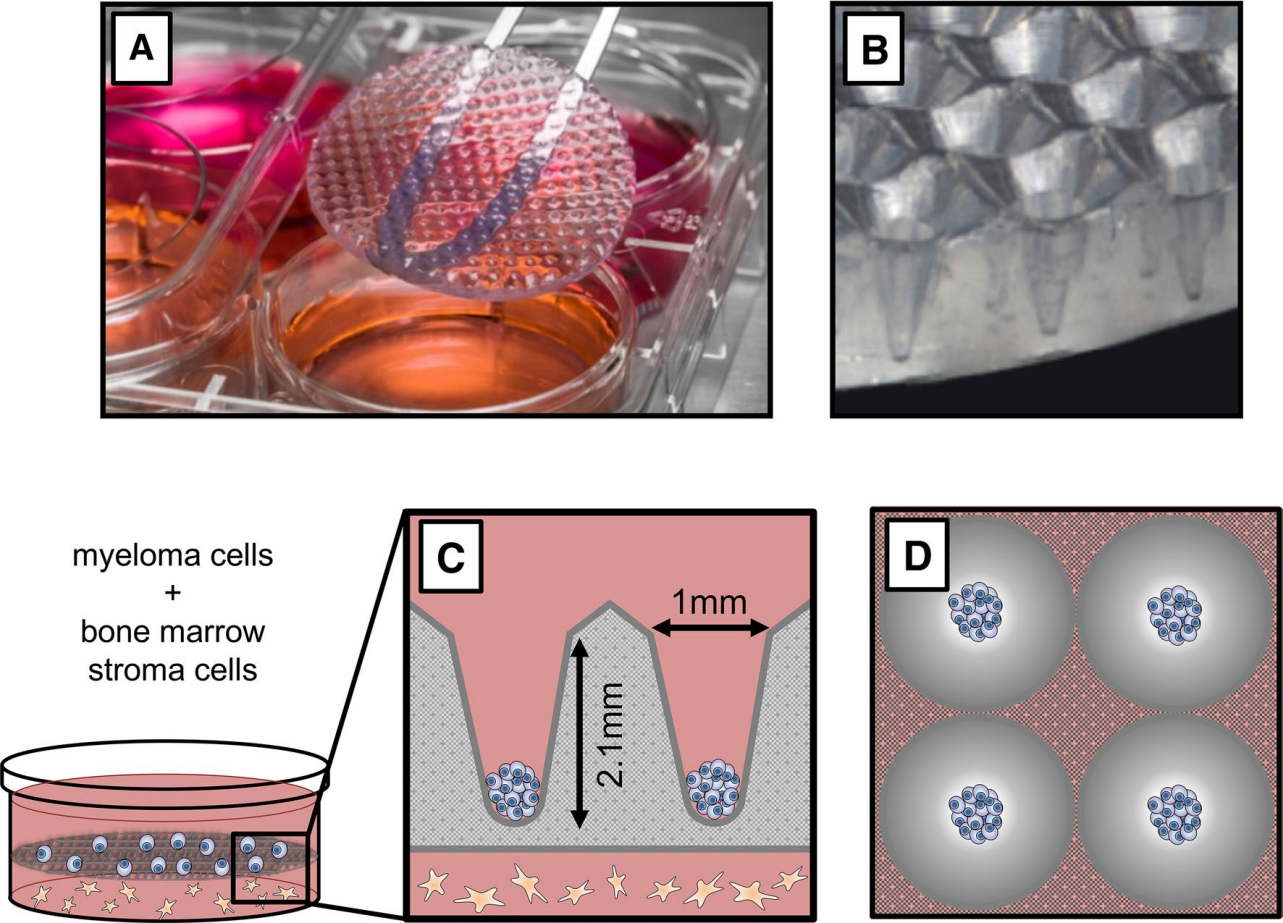
Details of the conical agarose microwell array platform. **A** Photography of the agarose microwell disk inserted into a 6-well plate. **B** Lateral view showing conical microwells inside a membrane. **C** Sketch depicting distance co-culture. MM cells are seeded into 3D CoSeedis^™^, and then, the microwell array is placed on the monolayer composed of BM stromal cells. Agarose permeability allows for diffusion of gas and small biomolecules. Each cavity measures 1 × 2.1 mm. **D** Sketch showing cell aggregates in four microwells

3D CoSeedis^™^ (abc biopply) is an agarose-based microwell device manufactured by computer-aided design (Fig. 1A) (Thomsen et al. 2017). The device is disk-shaped and contains conical microwells at a periodicity of 1 or 2 mm for 6- and 24-well plates, respectively (Fig. 1B). To monitor cell growth, plates were scanned on a high-resolution transmitted-light scanner (CanoScan 9000F Mark II) at 1200 dpi (Fig. 1C, D). From the central portion of each scan, an area of 4 cm^2^ comprising 100 or 400 microwells was cropped. Each aggregate was measured using ImageJ processing software (National Institute of Health). Quantification of cytokines was performed using the LUNARIS™ Human Multi-Plex Cytokine Kit (Ayoxxa Biosystems).

### Liquid overlay model

Liquid overlay refers to a cell culture model that allows for the reproducible formation of spheroidal cell clusters in vitro (Costa et al. 2014). In brief, all wells of a 96-well plate were coated with 50 μl of hot 1.5% autoclaved agarose (95 °C). Next, cells were seeded into the cavities and the plate was moved to an incubator at 37 °C with CO2 for at least 24 h to ensure the formation of consistent clusters in all wells.

### Retroviral transduction and selection of RPMI-8226 expressing histone H2B- mCherry

pMXs H2B-mCherry IRES blasticidin was established as previously described (Schnerch et al. 2013). 293 T cells were incubated for 10 h in the presence of calcium phosphate-precipitated plasmid DNA and 10 mM chloroquine. Virus-containing supernatant was collected twice (after 24 and 48 h). For transduction, RPMI-8226 cells were incubated in 4 ml of virus-containing supernatant supplemented with 2 ml of fresh growth medium and 5 μg/ml hexadimethrine bromide. Transduced RPMI-8226 were cultured in the presence of 20 μg/ml blasticidin to select for cells expressing histone H2B-mCherry.

### Confocal microscopy

3D microarrays containing mCherry-transduced RPMI-8226 were stained with equal amounts of CellTrace CFSE or CellTrace Violet (1.5 μl) for 30 min and washed twice. Confocal microscopy was performed on an LSM 880 Airyscan (Zeiss Microsystems).

### IHC staining

Microwell devices were incubated in 2% formalin solution overnight and sealed until further processing. Embedding was performed using 2.4% low melting point agarose. Following dehydration and paraffin embedding, 2 μm-thick sections were cut and mounted onto slides. Antibodies used for staining are displayed in Table S1.

### Cytotoxicity analysis

A total of 2 ml of HS-5 stroma cells were plated out with a density of 1 × 10^5^ cells per mL on the bottom of a 6-well plate. To equilibrate the CoSeedis™ matrix, the scaffold was set on top of the stroma layer for 2–3 h. After 24 h, media was replaced by 9 mL of MMCL-containing cell suspension (5 × 10^5^ cells per mL). Cells settled for another 24 h before either DMSO-diluted bortezomib (Selleck Chemicals) or auranofin (Santa Cruz) was added. After 48 h of drug exposure, cell viability was estimated using the CellTiter-Glo assay (Promega).

### Flow cytometry

Adherent cells were detached using trypsin–EDTA and transferred to filter tubes. In 3 ml fresh and cold PBS, cells were washed and centrifuged at 1200 rpm over 8 min. Pellets were dissolved and stained in 200 µL staining solution with antibodies as indicated in Table S1. 1 × 10^5^ cells were measured per condition on an FACS Calibur (Becton Dickinson). Analysis was performed using CellQuest^™^ Pro, FACS Diva (version 8.0.1) and FlowJo (version 10.3).

## Results

### 3D microwell design allows for aggregate formation and recapitulates BM support

**Fig. 2:**
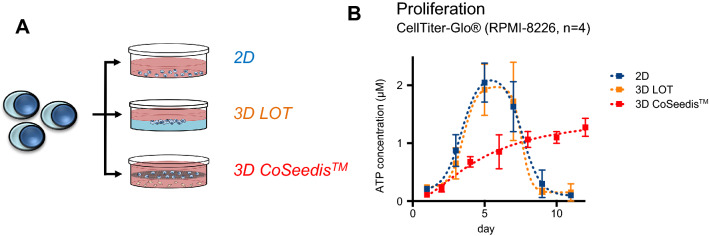
Proliferation in the conical agarose microwell array. **A** Sketch depicting the experimental approach. Proliferation of RPMI-8226 was assessed in a flat two-dimensional (2D) monolayer as compared to liquid overlay technique (LOT) versus the 3D conical agarose microwell array (3D CoSeedis^™^) platform. **B** ATP content indicates a bell-shaped growth pattern for RPMI-8226 cells in the 2D and LOT model. Cell growth in the 3D microwell model was delayed but followed a sigmoidal pattern and was ongoing after 12 days of observation

**Fig. 3:**
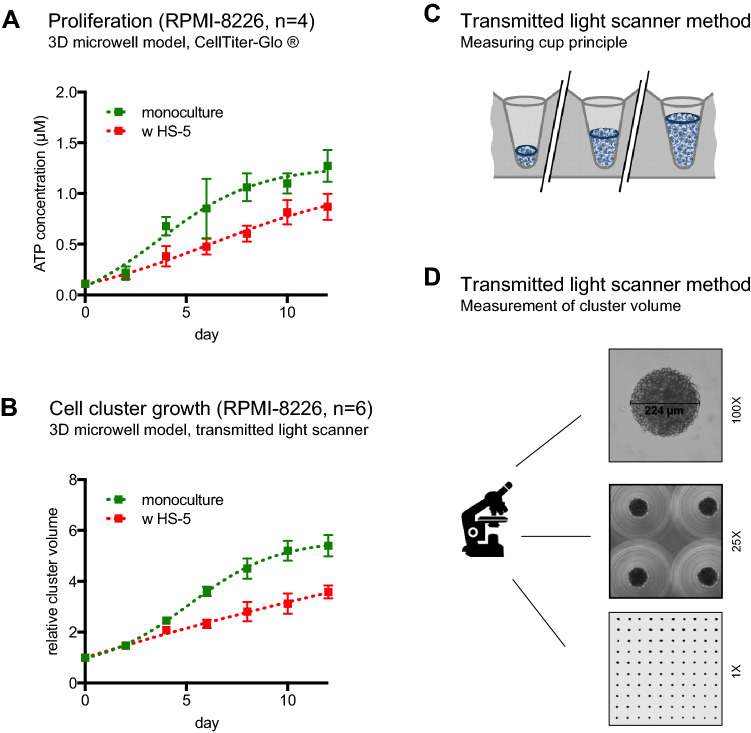
Impact of HS-5 stromal co-culture on proliferation and cluster size. **A** ATP content in RPMI-8226 cells culture in the 3D conical agarose microwell array as mono- vs. HS-5 co-culture. **B** Cluster volume of RPMI-8226 cells culture in the 3D conical agarose microwell array as mono- vs. HS-5 co-culture measured by transmitted-light scanner method. **C** Sketch showing cell aggregates in three microwells. This model uses the *measuring cup* principle for determination of cell proliferation. **D** Microscopy and scan of agarose matrix disk containing cell aggregates (100 × , 25 ×  and 1 × )

To compare and optimize ex vivo propagation of MM cells, RPMI-8226 cells were cultured in three different in vitro platforms. These platforms included a conventional 2D monolayer model, a liquid overlay approach, as well as the culture of cells in a novel 3D microwell device that allows for self-assembly of cell clusters (Figs. 1A–D, 2A). Aggregate formation in the latter model was additionally supported by a centrifugation step which forced seeding of MMCL into the cavities at an angle of 20°. Cell harvesting was performed by spinning the flipped matrices at a speed of 500 rpm. Cell proliferation in the monolayer and liquid overlay (measured as ATP content) followed bell-shaped kinetics with a maximal growth at ~ day 6, followed by a decay and full apoptosis at ~ day 10 (Fig. 2B). Cell proliferation in the microwell model corresponded to a sigmoidal curve, i.e., the initial growth was delayed but developed more durably and was ongoing after 12 days of observation. Since RPMI-8226 cells grow mostly in suspension, but may occasionally adhere and form clusters, microscopical inspection was performed throughout the experiment. Neither the monolayer nor the liquid overlay model produced cluster formation, whereas the cells in the microwells started to assemble and form instant cell aggregates within the conical microwells. As a second readout of cell proliferation, quantification of cell aggregate volumes over time was performed by a transmitted-light scanner approach (Thomsen et al. 2017). The proliferation rates determined by this approach faithfully recapitulated the data gained by the ATP-based quantification method (Fig. 3A, B). Both ATP quantification and transmitted-light scanner method revealed a slower proliferation rate for RPMI-8226 in the presence of the human BM stromal cell line compared to monoculture, which may point to a delayed but more durable establishment of cell–cell communication by continuous cytokine secretion (Fig. 3A, B).

**Fig. 4:**
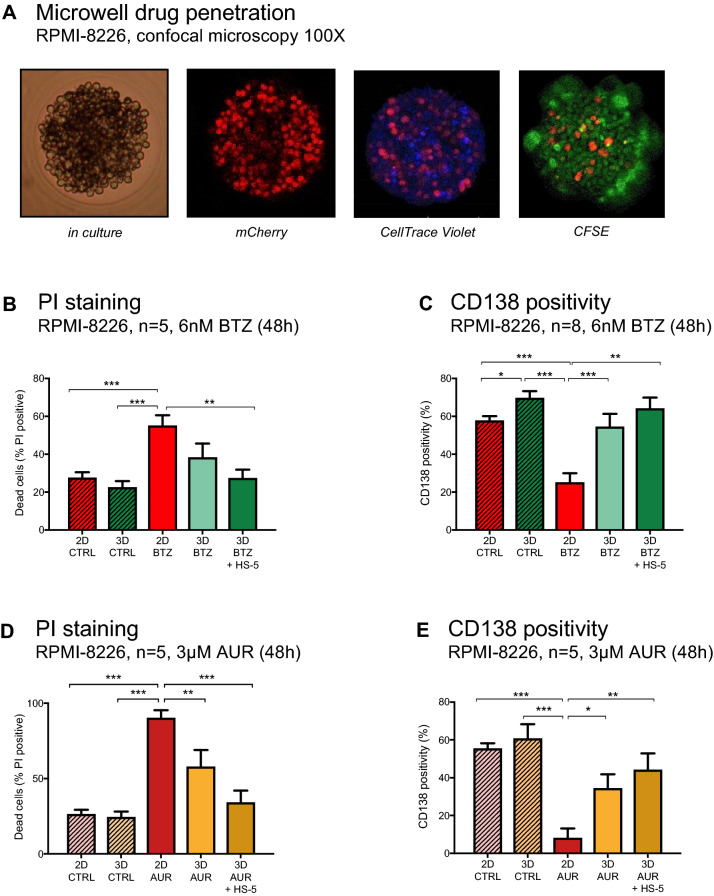
Drug resistance in the conical agarose microwell array. **A** Microscopy (left panel) and confocal microscopy of one microwell show equal distribution of CFSE (green) and CellTrace Violet within an aggregate of mCherry-transduced RPMI-8226. **B** Comparison of PI positivity in untreated RPMI-8226 cells and RPMI-8226 cells treated with bortezomib (6 nM) over 48 h. **C** Comparison of CD138 positivity in untreated RPMI-8226 cells and RPMI-8226 cells treated with bortezomib (6 nM) over 48 h. **D** Comparison of PI positivity in untreated RPMI-8226 cells and RPMI-8226 cells treated with auranofin (3 µM) over 48 h. **E** Comparison of CD138 positivity in untreated RPMI-8226 cells and RPMI-8226 cells treated with auranofin (3 µM) over 48 h. Conditions in B–E varied by model (2D vs. 3D microwell) and co-culture (monoculture vs. HS-5 co-culture). P values are as indicated *P ≤ 0.05, **P ≤ 0.01, ***P ≤ 0.001

To determine the permeability of our approach for cytokines and therapeutics, RPMI-8226 cells were transduced with the gene coding for the fluorescent protein mCherry followed by subsequent incubation with CellTrace CFSE and CellTrace Violet. Fluorescence was examined by confocal microscopy. Both dyes permeated the cell membrane of viable cells and penetrated to the core of each cell aggregate (Fig. 4A). The agarose hydrogel itself did not absorb any fluorescent dye, thereby permitting a transparent background for imaging analysis.

### Stromal co-culture and cluster formation foster resistance to bortezomib and auranofin

Based on previous reports describing the critical role of stromal support for drug resistance in MM (Azab et al. 2009a, b, 2012; Waldschmidt et al. 2017), we hypothesized that the addition of a distant co-culture support would provide further benefit for MM cells within the microwell cavities and therefore tested the sensitivity of MM cells toward chemotherapy in this system.

After 48 h, bortezomib-induced cytotoxicity in RPMI-8226 cells was ~ twofold lower in the 3D model as compared to the normal 2D flat culture (Fig. 4B), thereby demonstrating the impact of cluster formation on drug resistance of MM cells. Notably, sensitivity to bortezomib was even lower in the 3D model with HS-5 stromal support (*P* ≤ 0.01). To determine that reduced sensitivity to anti-MM drugs was not restricted to proteasome inhibitors, we recapitulated the impact of cluster formation and HS-5 stromal support by investigating the cytotoxic potency of the DUB inhibitor auranofin (AUR). AUR exemplifies a cytotoxic drug with potent in vitro activity in multiple cancers but so far limited efficacy in the clinic (Bonolo de Campos et al. 2020). As an inhibitor of the deubiquitinase system, AUR has reportedly high efficacy in diseases with a dysregulated ubiquitin–proteasome system (UPS), including MM (Nakaya et al. 2011; Raninga et al. 2015; Wang et al. 2019; Sze et al. 2020). The sensitivity to AUR in each model was exemplified by exposing MM cells to 3 µM AUR. Cytotoxicity after 48 h of AUR treatment was reduced by ~ 1.5-fold in RPMI-8226 cells treated within the 3D model as compared to cells treated within the normal 2D flat culture (Fig. 4D). Sensitivity to AUR was even lower in the 3D model with HS-5 stromal support (*P* ≤ 0.001). To determine that reduced sensitivity to AUR was not restricted to RPMI-8226, we recapitulated the impact of cluster formation and HS-5 stromal support by investigating the sensitivity to auranofin (AUR) in U266 as a second MMCL (Figure S1A) (Bonolo de Campos et al. 2020). CD138 positivity correlated with the cytotoxic potency of both drugs (Fig. 4C, E, S1B), with untreated cells showing significantly higher CD138 expression in the 3D vs. 2D setting. This observation may be critical for future phenotypic analyses as a more realistic approximation of antigen expression ex vivo may impact the evaluation of therapeutically relevant antigen expression.

In summary, our data propose a need to incorporate stromal co-culture into ex vivo modeling and demonstrate the superiority of our 3D versus 2D approaches.

### Propagation of primary MM cells is reproducible ex vivo

**Fig. 5:**
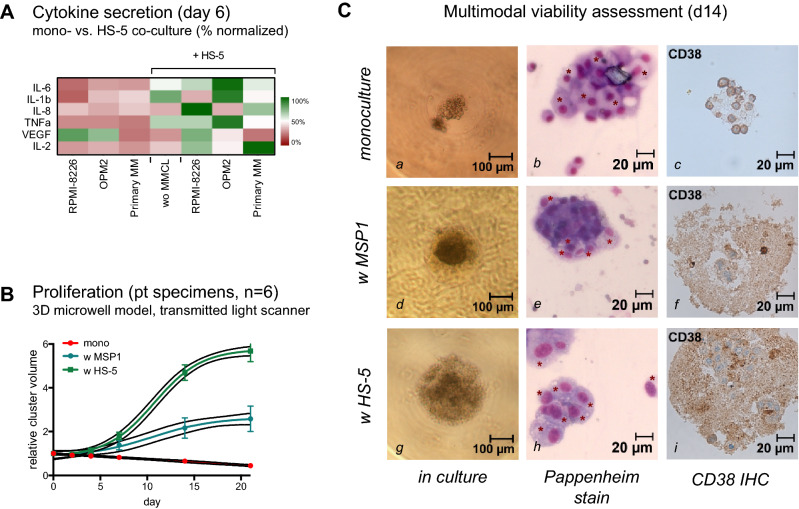
Long-term propagation of primary MM cells ex vivo. **A** Cytokine secretion measured by multiplex array from supernatants of RPMI-8226, OPM-2, and primary MM cells after 6 days with and without HS-5 co-culture. **B** Cluster volume measured by transmitted-light scanner methods shows the critical impact of co-culture for the propagation of primary patient specimens ex vivo (*n *= 6). Growth support is improved with HS-5 as compared to MSP-1. Cells continue to proliferate at day 21 ex vivo. **C** Longitudinal monitoring of primary MM cells derived from the bone marrow of a 51-year-old patient with high-risk IgG kappa MM. Assessment is shown for day 14 ex vivo using microscopy (left column), Pappenheim stain (middle panel) and IHC for CD38 (right panel). Serial monitoring demonstrated less cluster cell expansion of MM cells in monoculture (**a**). Plasma cell (PC) morphology via microscopy/Pappenheim stain remained apparent (**b**), but with few CD38 positive PCs in IHC (**c**). Co-culture with MSP-1 stimulated cluster expansion of primary PCs (**d**). PCs were densely accumulating (**e**) and led to much larger PC clusters with CD38 positive cells (**f**) in the presence of MSP-1 stromal support. Cluster size and CD38 positivity were further enhanced in **g**–**i** with HS-5 stromal support in 3D culture

To evaluate the applicability of our 3D microwell model for ex vivo propagation of even more relevant primary MM cells, we measured cytokine concentrations in the respective supernatants after 6 days of culture using RPMI-8226, OPM-2, and primary MM cells. We observed increased secretion of IL1-beta, IL-2, IL-6, IL-8, and TNF-alpha, but reduced VEGF production in the presence of HS-5 stromal cells (Fig. 5A, Table S2).

Cell growth for MM cells from primary BM aspirates was next assessed in the presence or absence of stromal cell support. Primary MM cells were derived from the BM of six RRMM patients with reported BM infiltration of CD138 + /CD38 + plasma cells ≥ 80% (Table 1). Growth monitoring over time revealed that primary MM cells in monoculture did not expand and showed a moderate linear decline in cell volume over the first 21 days of culture (Fig. 5B). Co-culture with MSP-1, an MM-derived human stromal cell line, induced a sigmoidal expansion, characterized by a > twofold increase in cell volume at day 21 of culture. HS-5 co-culture induced an even more beneficial increase in cell volume by > 5.5-fold after 3 weeks. Viability was continuously assessed and confirmed by microscopy, panchromatic Pappenheim staining, and immunohistochemical (IHC) staining for CD38 (Fig. 5C).

**Table 1:** Individual patient characteristics and summary

#	MM subtype	BM infiltration	CG	Age (yrs)	Gender	Status	ISS	Comment
1	IgA kappa	80%	HR	69	Female	RRMM	1	MM
2	IgG kappa	80%	HR	70	Male	RRMM	2	MM
3	kappa-LC	80%	HR	52	Male	RRMM	1	PCL
4	IgG kappa	80%	HR	83	Male	ID	3	MM
5	IgA kappa	90%	HR	66	Male	RRMM	3	MM
6	IgG kappa	90%	HR	51	Male	ID	3	MM
Σ median (range)	IgG (*n *= 3)	80% (80–90%)	HR (*n *= 6)	68 (51–83)	Female (*n *= 1)	RRMM (*n *= 6)	1 (*n *= 2)	MM (*n *= 5)
	IgA (*n *= 2)				Male (*n *= 5)	ID (*n *= 2)	2 (*n *= 1)	PCL (*n *= 1)
	kappa-LC (*n *= 1)						3 (*n *= 3)	

*MM* multiple myeloma, *RRMM* relapsed/refractory MM, *ID* initial diagnosis, *PCL* plasma cell leukemia, *EM-MM* extramedullary MM, *CG* cytogenetics, *HR* unfavorable cytogenetics defined as deletion 17p, t(4;14), t(14;16), t(14;20) or gain 1q

This demonstrated that our 3D microwell array is a suitable model to enable the survival and long-term culture of primary MM specimens ex vivo.

## Discussion

Treatment of MM has undergone substantial advances with the establishment of several FDA-approved agents with high clinical efficacy over the past decades. However, preclinical models for MM remain inefficient in predicting later clinical efficacy. Between 1961 and 2013, single agent activity has been reported for more than 400 preclinically tested compounds, but only ten of these agents could be validated in phase III trials and are nowadays approved treatment options for the clinical management of patients (Kortuem et al. 2014). Understanding preclinical modeling for MM may help to optimize the translation of in vitro findings into clinical use, but is unfortunately still limited by the inability to reproducibly propagate primary MM patient specimens ex vivo.

MM cells strongly depend on the interaction with their BM microenvironment (Azab et al. 2009b; Waldschmidt et al. 2017). Current in vitro models tend to overemphasize anti-MM effects as they typically do not simulate such interaction of MM cells with their protective niche. As our knowledge on MM and its microenvironment has dramatically increased over the last years, more complex in vitro models have been introduced, thereby providing a wider range of test systems from low- to high-throughput approaches (Schüler et al. 2013). These platforms include two-dimensional (2D) monolayers, bioreactors, scaffold-based models, and liquid overlay cultures (Abe et al. 2004; Kirshner et al. 2008; Zdzisińska et al. 2009; Breslin and O’Driscoll 2013; Reagan et al. 2014; de la Puente et al. 2015; Jakubikova et al. 2016; Santo et al. 2017). 2D models allow for high-throughput drug screening, but do not adequately assess the interaction of MM cells with their environment. More sophisticated 3D co-culture systems have integrated additional components which permit evaluation of a broader range of therapeutic strategies, but increase the complexity and thereby limit the applicability of 3D models to smaller throughput series (Table S3) (Breslin and O’Driscoll 2013; Schüler et al. 2013; Santo et al. 2017).

While prior models have reported that most primary MM cells do not proliferate ex vivo (Drewinko et al. 1981; Robillard et al. 2005), we here report on a novel conical agarose 3D microwell platform for the reproducible culture of primary MM cells. Our agarose-based microwell design facilitates aggregate formation which in turn has a significant impact on the long-term viability of BM-derived primary MM cells ex vivo. We recapitulate that stromal co-culture is essential for in vitro modeling of drug resistance phenomena and adequate preclinical prediction of drug sensitivity. To examine the applicability of our model for ex vivo drug testing, we investigated the cytotoxic effect of bortezomib and the DUB inhibitor auranofin with reportedly high in vitro efficacy in primary MM samples (Bonolo de Campos et al. 2020). We show that while both bortezomib and auranofin were highly potent in the absence of stromal cell support, they induced less cytotoxicity if MM cells (i) form cell–cell aggregates, and (ii) receive cytokine support via HS-5 stromal co-culture. This finding in the 3D co-culture setting is in line with prior work from our group and others describing stroma-induced drug resistance under 2D co-culture conditions (Azab et al. 2009b; Waldschmidt et al. 2017). At the same time, our platform allows for substantially longer cultivation periods of primary patient material and for robust separation of target cell populations, this being highly relevant for drug screening and testing over more extended cultivation periods (> 2–3 days) as currently feasible with 2D culture systems (Zlei et al. 2007; Udi et al. 2013; Lorenz et al. 2016).

Primary MM cells benefitted from higher cytokine levels in the supernatant and could be maintained in culture up to a culture period of 21 days. We have previously shown that combined supplementation of cytokines (IL-6, IGF-1, CXCL12, Galectin-1, and IL-1a) has substantial impact on the growth of MM cells (Zlei et al. 2007). Here, we provide more detailed cytokine characterization in the 3D setting, and show that IL-6, IL-1b, IL-8, and TNFα secretion seem to be stimulated by the interaction of MMCL and primary MM cells with their respective stromal co-culture partner. This observation is in line with Zdzisinska and colleagues who reported increased IL-11 and HGF secretion by MM-derived mesenchymal stem cells (MSCs) in the 3D versus 2D co-culture setting (Zdzisińska et al. 2009). Other than expected, addition of HS-5 led to a decrease of VEGF in the co-culture supernatants. This appeared different to our current understanding on the role of neo-angiogenesis and VEGF signaling for MM cell propagation (Le Gouill et al. 2004; Neri et al. 2011) and most likely reflected the more delayed growth kinetics in our model. Studies with serial cytokine measurements over time are currently ongoing and will investigate if incorporation of other cell types, most critically endothelial cells, may provide an even better cytokine environment for the long-term propagation of primary MM cells (Podar and Anderson 2007).

Aside from our platform, other in vitro models have been developed with the intent to better recapitulate the MM-BM niche: osteoclasts, murine stromal cell lines, immature dendritic cells, and mesenchymal stem cells have all proven advantageous to stimulate proliferation of MM cells in vitro (Chauhan et al. 1996; Abe et al. 2004; Li et al. 2008; Kirshner et al. 2008; Azab et al. 2012). Major challenges of these currently available co-culture platforms, however, remain to (i) guarantee reproducible growth characteristics and (ii) avoid long-term phenotypic modulation attributable to the models themselves (Zlei et al. 2007; Yaccoby 2010; Udi et al. 2013).

By providing a 3D scaffold with preformed cavities, our model allows for the reproducible ex vivo propagation of MMCL and especially primary MM cells. This platform incorporates co-culture partners from the BM niche which seems critical to realistically determine the efficacy of future therapeutic compounds. Although it remains to be seen whether 3D models can substitute conventional testing systems, they may open up an entirely new frame for the in vitro assessment of anti-MM targets (Schüler et al. 2013). From our current understanding, we propose that 2D and 3D co-culture models should ideally complement each other with 2D being used for short-term (< 2–3 days) assessment of MMCL in vitro and 3D approaches to be preferred for ex vivo cultivation of primary material over longer periods.

In summary, we here report on a novel 3D in vitro platform for the preclinical propagation of primary MM cells. While the readout of our system focuses primarily on morphological changes, cell viability, and cell growth kinetics, it also allows for the evaluation of surface marker expression, migration, and target expression of various anti-MM agents. From a clinical viewpoint, this model may improve our ability to better predict later clinical success of preclinically active compounds to reduce time and financial expenses of drug discovery for MM at the earliest stage possible.

## Supplementary Information

Below is the link to the electronic supplementary material.

Supplementary file1 (PDF 51 KB)

Supplementary file2 (DOCX 211 KB)

## Data Availability

Not applicable.

## References

[CR1] Abe M, Hiura K, Wilde J et al (2004) Osteoclasts enhance myeloma cell growth and survival via cell-cell contact: a vicious cycle between bone destruction and myeloma expansion. Blood 104:2484–2491. 10.1182/blood-2003-11-3839 15187021 10.1182/blood-2003-11-3839

[CR2] American Cancer Society. Key statistics for multiple myeloma. https://www.cancer.org/cancer/multiple-myeloma/about/key-statistics.html. Accessed 13 June 2021

[CR3] Azab AK, Azab F, Blotta S et al (2009a) RhoA and Rac1 GTPases play major and differential roles in stromal cell-derived factor-1-induced cell adhesion and chemotaxis in multiple myeloma. Blood 114:619–629. 10.1182/blood-2009-01-199281 19443661 10.1182/blood-2009-01-199281 PMC2713475

[CR4] Azab AK, Runnels JM, Pitsillides C et al (2009b) CXCR4 inhibitor AMD3100 disrupts the interaction of multiple myeloma cells with the bone marrow microenvironment and enhances their sensitivity to therapy. Blood 113:4341–4351. 10.1182/blood-2008-10-186668 19139079 10.1182/blood-2008-10-186668 PMC2676090

[CR5] Azab AK, Hu J, Quang P et al (2012) Hypoxia promotes dissemination of multiple myeloma through acquisition of epithelial to mesenchymal transition-like features. Blood 119:5782–5794. 10.1182/blood-2011-09-380410 22394600 10.1182/blood-2011-09-380410 PMC3382938

[CR6] Bonolo de Campos C, Meurice N, Petit JL et al (2020) “Direct to Drug” screening as a precision medicine tool in multiple myeloma. Blood Cancer J 10:54. 10.1038/s41408-020-0320-7 32393731 10.1038/s41408-020-0320-7 PMC7214452

[CR7] Breslin S, O’Driscoll L (2013) Three-dimensional cell culture: the missing link in drug discovery. Drug Discov Today 18:240–249. 10.1016/j.drudis.2012.10.003 23073387 10.1016/j.drudis.2012.10.003

[CR8] Chauhan D, Uchiyama H, Akbarali Y et al (1996) Multiple myeloma cell adhesion-induced interleukin-6 expression in bone marrow stromal cells involves activation of NF-kappa B. Blood 87:1104–1112 8562936

[CR9] Costa EC, Gaspar VM, Coutinho P, Correia IJ (2014) Optimization of liquid overlay technique to formulate heterogenic 3D co-cultures models. Biotechnol Bioeng 111:1672–1685. 10.1002/bit.25210 24615162 10.1002/bit.25210

[CR10] de la Puente P, Muz B, Gilson RC et al (2015) 3D tissue-engineered bone marrow as a novel model to study pathophysiology and drug resistance in multiple myeloma. Biomaterials 73:70–84. 10.1016/j.biomaterials.2015.09.017 26402156 10.1016/j.biomaterials.2015.09.017 PMC4917006

[CR11] de la Puente P, Quan N, Hoo RS et al (2016) Newly established myeloma-derived stromal cell line MSP-1 supports multiple myeloma proliferation, migration, and adhesion and induces drug resistance more than normal-derived stroma. Haematologica 101:e307-311. 10.3324/haematol.2016.142190 27081175 10.3324/haematol.2016.142190 PMC5004479

[CR12] Drewinko B, Alexanian R, Boyer H et al (1981) The growth fraction of human myeloma cells. Blood 57:333–338 7448427

[CR13] Frigyesi I, Adolfsson J, Ali M et al (2014) Robust isolation of malignant plasma cells in multiple myeloma. Blood 123:1336–1340. 10.1182/blood-2013-09-529800 24385542 10.1182/blood-2013-09-529800

[CR14] Ghobrial IM, Liu C-J, Redd RA et al (2020) A phase Ib/II trial of the first-in-class anti-CXCR4 antibody ulocuplumab in combination with lenalidomide or bortezomib plus dexamethasone in relapsed multiple myeloma. Clin Cancer Res 26:344–353. 10.1158/1078-0432.CCR-19-0647 31672767 10.1158/1078-0432.CCR-19-0647 PMC11753616

[CR15] Jakubikova J, Cholujova D, Hideshima T et al (2016) A novel 3D mesenchymal stem cell model of the multiple myeloma bone marrow niche: biologic and clinical applications. Oncotarget 7:77326–77341. 10.18632/oncotarget.12643 27764795 10.18632/oncotarget.12643 PMC5357212

[CR16] Kirshner J, Thulien KJ, Martin LD et al (2008) A unique three-dimensional model for evaluating the impact of therapy on multiple myeloma. Blood 112:2935–2945. 10.1182/blood-2008-02-142430 18535198 10.1182/blood-2008-02-142430

[CR17] Kortuem KM, Zidich K, Schuster SR et al (2014) Activity of 129 single-agent drugs in 228 phase I and II clinical trials in multiple myeloma. Clin Lymphoma Myeloma Leuk 14:284-290.e5. 10.1016/j.clml.2013.12.015 24565465 10.1016/j.clml.2013.12.015 PMC4092053

[CR18] Kyle RA, Rajkumar SV (2009) Criteria for diagnosis, staging, risk stratification and response assessment of multiple myeloma. Leukemia 23:3–9. 10.1038/leu.2008.291 18971951 10.1038/leu.2008.291 PMC2627786

[CR19] Le Gouill S, Podar K, Amiot M et al (2004) VEGF induces Mcl-1 up-regulation and protects multiple myeloma cells against apoptosis. Blood 104:2886–2892. 10.1182/blood-2004-05-1760 15217829 10.1182/blood-2004-05-1760

[CR20] Li X, Pennisi A, Yaccoby S (2008) Role of decorin in the antimyeloma effects of osteoblasts. Blood 112:159–168. 10.1182/blood-2007-11-124164 18436739 10.1182/blood-2007-11-124164 PMC2435686

[CR21] Lorenz J, Waldschmidt J, Wider D et al (2016) From CLL to multiple myeloma - spleen tyrosine kinase (SYK) influences multiple myeloma cell survival and migration. Br J Haematol 174:985–989. 10.1111/bjh.13825 26491948 10.1111/bjh.13825

[CR22] Nakaya A, Sagawa M, Muto A et al (2011) The gold compound auranofin induces apoptosis of human multiple myeloma cells through both down-regulation of STAT3 and inhibition of NF-κB activity. Leuk Res 35:243–249. 10.1016/j.leukres.2010.05.011 20542334 10.1016/j.leukres.2010.05.011

[CR23] Neri P, Ren L, Azab AK et al (2011) Integrin β7-mediated regulation of multiple myeloma cell adhesion, migration, and invasion. Blood 117:6202–6213. 10.1182/blood-2010-06-292243 21474670 10.1182/blood-2010-06-292243 PMC3122944

[CR24] Podar K, Anderson KC (2007) Inhibition of VEGF signaling pathways in multiple myeloma and other malignancies. Cell Cycle 6:538–542. 10.4161/cc.6.5.3922 17351339 10.4161/cc.6.5.3922

[CR25] Raje NS, Faber EA, Richardson PG et al (2016) Phase 1 study of tabalumab, a human anti-B-cell activating factor antibody, and bortezomib in patients with relapsed/refractory multiple myeloma. Clin Cancer Res 22:5688–5695. 10.1158/1078-0432.CCR-16-0201 27287072 10.1158/1078-0432.CCR-16-0201

[CR26] Raje NS, Moreau P, Terpos E et al (2017) Phase 2 study of tabalumab, a human anti-B-cell activating factor antibody, with bortezomib and dexamethasone in patients with previously treated multiple myeloma. Br J Haematol 176:783–795. 10.1111/bjh.14483 28005265 10.1111/bjh.14483

[CR27] Raninga PV, Di Trapani G, Vuckovic S et al (2015) Inhibition of thioredoxin 1 leads to apoptosis in drug-resistant multiple myeloma. Oncotarget 6:15410–15424. 10.18632/oncotarget.3795 25945832 10.18632/oncotarget.3795 PMC4558160

[CR28] Reagan MR, Mishima Y, Glavey SV et al (2014) Investigating osteogenic differentiation in multiple myeloma using a novel 3D bone marrow niche model. Blood 124:3250–3259. 10.1182/blood-2014-02-558007 25205118 10.1182/blood-2014-02-558007 PMC4239334

[CR29] Robillard N, Pellat-Deceunynck C, Bataille R (2005) Phenotypic characterization of the human myeloma cell growth fraction. Blood 105:4845–4848. 10.1182/blood-2004-12-4700 15741217 10.1182/blood-2004-12-4700

[CR30] Santo VE, Rebelo SP, Estrada MF et al (2017) Drug screening in 3D in vitro tumor models: overcoming current pitfalls of efficacy read-outs. Biotechnol J. 10.1002/biot.201600505 27966285 10.1002/biot.201600505

[CR31] Schmidt-Hieber M, Pérez-Andrés M, Paiva B et al (2011) CD117 expression in gammopathies is associated with an altered maturation of the myeloid and lymphoid hematopoietic cell compartments and favorable disease features. Haematologica 96:328–332. 10.3324/haematol.2010.031872 20971816 10.3324/haematol.2010.031872 PMC3031704

[CR32] Schnerch D, Follo M, Felthaus J et al (2013) The 3’ untranslated region of the cyclin B mRNA is not sufficient to enhance the synthesis of cyclin B during a mitotic block in human cells. PLoS ONE 8:e74379. 10.1371/journal.pone.0074379 24058555 10.1371/journal.pone.0074379 PMC3772928

[CR33] Schüler J, Ewerth D, Waldschmidt J et al (2013) Preclinical models of multiple myeloma: a critical appraisal. Expert Opin Biol Ther 13(Suppl 1):S111-123. 10.1517/14712598.2013.799131 23742200 10.1517/14712598.2013.799131

[CR34] Sze JH, Raninga PV, Nakamura K et al (2020) Anticancer activity of a Gold(I) phosphine thioredoxin reductase inhibitor in multiple myeloma. Redox Biol 28:101310. 10.1016/j.redox.2019.101310 31514052 10.1016/j.redox.2019.101310 PMC6742860

[CR35] Thomsen AR, Aldrian C, Bronsert P et al (2017) A deep conical agarose microwell array for adhesion independent three-dimensional cell culture and dynamic volume measurement. Lab Chip 18:179–189. 10.1039/c7lc00832e 29211089 10.1039/c7lc00832e

[CR36] Udi J et al (2013) Potent in vitro and in vivo activity of sorafenib in multiple myeloma: induction of cell death, CD138-downregulation and inhibition of migration through actin depolymerization. In: Br J Haematol. https://pubmed.ncbi.nlm.nih.gov/23384035/. Accessed 5 Feb 2021 10.1111/bjh.12226 23384035

[CR37] Varga C, Waldschmidt JM, Gandolfi S, Richardson PG (2020) Current antibody-based therapies for the treatment of multiple myeloma. Clin Adv Hematol Oncol HO 18:736–748 33406065

[CR38] Voorhees PM, Manges RF, Sonneveld P et al (2013) A phase 2 multicentre study of siltuximab, an anti-interleukin-6 monoclonal antibody, in patients with relapsed or refractory multiple myeloma. Br J Haematol 161:357–366. 10.1111/bjh.12266 23432640 10.1111/bjh.12266 PMC5837861

[CR39] Waldschmidt JM, Simon A, Wider D et al (2017) CXCL12 and CXCR7 are relevant targets to reverse cell adhesion-mediated drug resistance in multiple myeloma. Br J Haematol 179:36–49. 10.1111/bjh.14807 28670693 10.1111/bjh.14807

[CR40] Wang J, Wang J, Lopez E et al (2019) Repurposing auranofin to treat TP53-mutated or PTEN-deleted refractory B-cell lymphoma. Blood Cancer J 9:95. 10.1038/s41408-019-0259-8 31780660 10.1038/s41408-019-0259-8 PMC6882812

[CR41] Yaccoby S (2010) Osteoblastogenesis and tumor growth in myeloma. Leuk Lymphoma 51:213–220. 10.3109/10428190903503438 20038269 10.3109/10428190903503438 PMC2849287

[CR42] Zdzisińska B, Roliński J, Piersiak T, Kandefer-Szerszeń M (2009) A comparison of cytokine production in 2-dimensional and 3-dimensional cultures of bone marrow stromal cells of multiple myeloma patients in response to RPMI8226 myeloma cells. Folia Histochem Cytobiol 47:69–74. 10.2478/v10042-009-0015-1 19419941 10.2478/v10042-009-0015-1

[CR43] Zlei M, Egert S, Wider D et al (2007) Characterization of in vitro growth of multiple myeloma cells. Exp Hematol 35:1550–1561. 10.1016/j.exphem.2007.06.016 17889722 10.1016/j.exphem.2007.06.016

