# Supplementary figures and images for: Ex vivo propagation in a novel 3D high-throughput co-culture system for multiple myeloma
Source: J Cancer Res Clin Oncol. 2022 Jan 24;148(5):1045–55. doi: 10.1007/s00432-021-03854-6 (PMC9016043; doi:10.1007/s00432-021-03854-6)

**A** PI staining (U266, n=5)  
3 $\mu$ M AUR (48h)

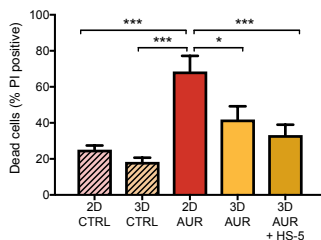

**B** CD138 positivity (U266, n=5)  
3 $\mu$ M AUR (48h)

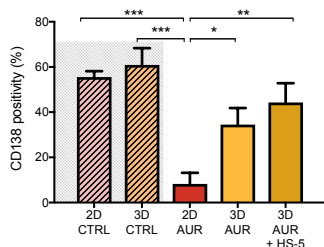

Figure S1

Supplement: Supplementary file 1 — Supplementary file1 (PDF 51 KB) [file 432_2021_3854_MOESM1_ESM.pdf]
