# Supplementary material for: Ex vivo propagation in a novel 3D high-throughput co-culture system for multiple myeloma
Source: J Cancer Res Clin Oncol. 2022 Jan 24;148(5):1045–55. doi: 10.1007/s00432-021-03854-6 (PMC9016043; doi:10.1007/s00432-021-03854-6)
Supplement: Supplementary file 2 — Supplementary file2 (DOCX 211 KB) [file 432_2021_3854_MOESM2_ESM.docx]

***Ex* *vivo* propagation in a novel high-throughput coculture system for multiple myeloma**

Supplementary data

**Original Paper**

**Journal of Cancer Research and Clinical Oncology**

**Table S1. Antibodies used for Western Blot, IHC and FACS experiments**

|  | **Target** | **Source** | **Dilution** | **Manufacturer** | **Clone** |
| --- | --- | --- | --- | --- | --- |
| **Primary antibodies (WB)** | ac-α-tubulin | mouse IgG2b | 1:1000 | Sigma Aldrich | 6-11B-1 |
|  | ac histone 3 | rabbit IgG | 1:1000 | Merck Millipore | polyclonal |
|  | ac histone 4 | rabbit IgG | 1:1000 | Abcam | EPR16606 |
|  | ß-actin | mouse IgG1 | 1:5000 | Sigma Aldrich | 15G5A11/E2 |
|  | GADPH | mouse IgG2b | 1:1000 | Biozol Diagnostica | GT239 |
| **Secondary antibodies (WB)** | mouse | IgG HRP-linked | 1:5000 | GeneTex | goat polyclonal IgG |
|  | rabbit | IgG HRP-linked | 1:5000 | GE Healthcare | donkey monoclonal IgG |
| **IHC antibodies** | CD38 | mouse IgG1 | NA | Leica Biosystems | SPC32 |
| **FACS antibodies** | CD38/ PerCP-Cy5.5 | NA | 1:40 | Biolegend | HIT2 |
|  | CD38/ PE-Cy7 | NA | 1:40 | Biolegend | HB-7 |
|  | CD138/ FITC | NA | 1:20 | Becton Dickinson | MI15 |
|  | SLAMF7/ PE-Cy7 | NA | 1:40 | Biolegend | 162.1 |
|  | Human BD FcBlock^TM^ | NA | 1:100 | Becton Dickinson | - |

**Table S2. Cytokine multiplex array for mono- vs. co-cultures (day 6, µg/mL)**

**Table S3. Advantages and limitations of current 3D co-culture models**

|  | **Advantages** | **Limitations** | **References** |
| --- | --- | --- | --- |
| CoSeedis | - easy handling - feasible imaging/ IHC - aggregate formation is reproducible - applicable for high-throughput screening - eligible for semi-adherent cells - distance co-culture | - limited variability - not applicable to mimic extracellular matrix | Thomsen et al. 2017 |
| Liquid overlay | - easy processing and imaging - reproducibility (eligible for high-throughput-screening) - cost efficiency | - not applicable to mimic extracellular matrix - limited variability | Costa et al. 2014  Costa et al. 2018 |
| Scaffold-based | - reflects spatial interaction with cytokines and adhesion molecules - contact co-culture - mimics extracellular matrix - IHC-eligible | - cost - more complex imaging - time-consuming - not applicable for high-throughput screening | Kirshner et al. 2008  Reagan et al. 2014  De la Puente et al. 2015 |
| Bioreactor | - allows formation of large spheroids - reflects dynamic drug diffusion | - technically challenging - only adherent cells - not applicable for high-throughput screening - more complex imaging - spheroid formation may be heterogeneous | Santo et al. 2017 |

**Figure S1. Auranofin drug resistance in the conical agarose microwell array**

**Figure S1. A.** Comparison of PI positivity in untreated U266 cells and U266 cells treated with auranofin (3µM) over 48 hours. **B.** Comparison of CD138 positivity in untreated U266 cells and U266 cells treated with auranofin (3µM) over 48 hours. Conditions in A-B varied by model (2D vs. 3D microwell) and co-culture (monoculture vs. HS-5 co-culture). P values are as indicated *P≤0.05, **P≤0.01, ***P≤0.001.
